# Effective practices for improving service professionals’ ethical behaviors: A multiple method study

**DOI:** 10.3389/fpsyg.2022.1042142

**Published:** 2022-12-07

**Authors:** Ying Hu, Yefei Yang, Peter K. C. Lee

**Affiliations:** ^1^Law School, Huazhong University of Science and Technology, Wuhan, China; ^2^Graduate School, Zhongnan University of Economics and Law, Wuhan, China; ^3^School of Economics and Management, Beijing Jiaotong University, Beijing, China; ^4^Research Center for Central and Eastern Europe, Beijing Jiaotong University, Beijing, China; ^5^Keele Business School, Keele University, Keele, United Kingdom

**Keywords:** behavior control, ethics, professional service organizations, role stress, multi-method

## Abstract

**Introduction:**

Enhancing frontline professional service employees’ ethics has been an increasingly important issue for organizations in sustaining their reputation and long-term profitability. While many organizations have implemented general ethics programes such as ethics codes and ethical training, unethical scandals regularly still appear in many service organizations. This research offers new insights into the practices that can effectively enhance marketing practitioners’ ethical behaviors and the pertinent contextual factors that have a bearing on the effectiveness of ethics programes.

**Methods:**

It uses a multi-method methodology to conduct two studies in the Chinese banking setting. Based on the rank of revenue and profitability published by Fortune magazine of year 2021, in Study 1, we choose five main Chinese banking organizations to conduct case studies to explore the framework of effective ethics programes of banks. In Study 2 we use the valid instruments from the literature to measure the involved constructs and employs data from randomly selected 146 frontline banking teams in five main Chinese banking organizations to examine the effectiveness of three specific ethics practices and ascertain the moderating role of role stress in such effectiveness.

**Results and discussion:**

Our findings indicate the effective behavior control practices within organizations’ ethics programes and the implications of having a stressful workplace when adopting such practices. In addition, we integrate organizational concepts regarding behavior control and employee ethics, and use two empirical methods to systematically explore the effectiveness of ethics programes. This paper advances the management and marketing literature and has significant managerial implications for improving frontline service professionals’ ethical behaviors.

## Introduction

Human morality involves many aspects, such as, people readily use the information about another’s morality when judging their competence and immoral behavior could be caused by work environment and performance evaluation, eventually affecting the outcomes of their daily activities (Stellar and Willer, 2018; Liu et al., 2021; Huang et al., 2022). In particular, the emergence of fiercely competitive business environments and the rise of a wide variety of publicity media are underscoring the rising importance of market practitioners’ ethics because it significantly affects an organization’s customer retention capability, corporate reputation, and financial performance (Erwin, 2011; Michaelson and Tosti-Kharas, 2019). There is some literature that focuses on organizational unethical behavior. With such behavior (e.g., occupy public resources for personal purposes), individuals intend to achieve some benefits from the organization, which directly harms to organizational benefits (Hong et al., 2021; Liu et al., 2021). Also, the literature proposes that formal and institutional documents (e.g., industry regulations or standards) and the implementation of practices such as ethical leadership in organizations can reduce unethical incidents (Erwin, 2011; Lehnert et al., 2016).

However, in service operations, frontline employees’ unethical behavior could bring the benefits to organizations and their own interests in short time, which, however, have more possibility to affect the development of the organizations negatively in long time (Hirschfeld and Van Scotter, 2018; Silveira, 2022; Wang et al., 2021). Such unethical behavior is difficult to be monitored in a short time, which leads that current formal and institution documents proposed by the literature seems to have limited role in reducing these behaviors. Indeed, the ethical scandals still regularly occur in many service organizations (Silveira, 2022). For example, Wells Fargo (an American multinational financial services company) fired 5,300 employees due to their unethical behaviors in creating millions of banks and credit card accounts that customers did not authorize (Auletto and Miller, 2007). GlaxoSmithKline PLC (a British pharmaceutical company) paid 3 billion dollars fine for some employees’ unethical and illegal marketing activities (Plumridge, 2014). China’s Minsheng Bank sold forged wealth management products to customers including elderlies, which caused investor protest and police investigations (Huang and Guo, 2017). Indeed, a survey from 217 large global companies demonstrated that observed unethical behaviors are keeping upward, although they have spent much money to reduce unethical behaviors (e.g., one million dollars invested in ethics program for every billion revenue) (Bazerman and Tenbrunsel, 2011). This evidence highlights a need for more relevant insights to help organizations to improve the effectiveness of their ethics programs, so enhancing their employees’ ethical behaviors.

Based on Merchant and Van der Stede (2012)’s framework, practices within ethics programs can be categorized into personnel and culture control practices (e.g., ethical training, ethics codes, ethical committee development etc.), action control practices (e.g., process control, auditing systems etc.), and result control practices (e.g., reward schemes, punishment approaches etc.). Indeed, the findings on the effectiveness of ethics programs are mixed and inconsistent. The National Business Ethics Survey (NBES) of 2013 showed that nearly 80% of surveyed organizations conducted culture control practices, e.g., providing ethical training and communicating internally about wrongdoing behaviors and 67% of them conducted action control practices, e.g., installing ethical performance evaluation system. This indicates that organizations have recognized the importance of ethics programs and begun to pay strategic attention to adopting pertinent practices for their ethics programs. Also, empirical studies have demonstrated that ethics programs (e.g., ethical training, principles of conduct and evaluation system etc.) can improve marketing practitioners’ ethical behaviors (Weaver et al., 1999; Somers, 2001). However, some findings in the 2013 NBES survey show that half of the organizations with ethics programs still perceived that unethical behaviors are very prevalent in their workplaces, and employees in organizations with ethics programs reported more unethical behaviors than those in organizations with no such programs in place. Thus, there are two gaps. (1) Indeed, most currently popular ethics programs for organizational unethical behavior have not been fully effective in positively inducing service employee ethical behaviors. (2) Such mixed and inconsistent could be due to the different components of the ethics programs that organizations have implemented and the different contingencies associated with their workplaces. To fill the gaps, our study takes into account the wide range of practices incorporated into the ethics programs and the leveraging roles of special characteristics of services operations.

Thus, we propose three research questions: (1) Whether the ethics programs of service organizations are effective in enhancing employee behaviors? (2) What are contextual characteristics to influence the effectiveness of such practices? (3) What is the specific relationship among the practices, contextual factors, and employee ethical behaviors? Revealing that organizations generally follow a range of control practices in their ethics programs, the NBES survey does not clarify which practices are more effective (Merchant and Van der Stede, 2012). Thus, we use empirical exploratory approach research methods for the first two research questions and use a large-scale survey for the last research question based on sufficient data. Therefore, we adopt a multi-method approach by collecting data from the banking industry of China.

Study 1 uses five main Chinese banking organizations based on the rank of revenue and profitability published by Fortune magazine of year 2021 to identify the potentially effective practices that can indeed enhance employee ethical behaviors in the context of this study and their relevant contextual factors. The findings highlight the effectiveness of action control practices within ethics programs and the contingent effects of several contextual characteristics. Study 2 seeks to develop a deeper understanding of the relationships among behavior control practices, employee ethical behaviors, and contextual factors in randomly selected 146 frontline banking teams from five main Chinese banking organizations. The use of a survey in Study 2 helps addressing the potential problem of limited generalizability of Study 1.

The contribution of this research lies in its connections of ethics programs with psychological characteristics of frontline employees (i.e., role stress) to explore how behavior control practices (i.e., risk control, process control formality, and information technology intensity) can take effect role in influencing employees’ ethical behaviors, which can enrich the ethical and psychological literature. Our findings not only add new insights to the relevant literature, but also provide useful managerial guidelines for professional organizations (in particular, banks) to enhance their ethical performance.

## Theoretical background

### The practices of ethics programs

Ethics programs are organizational control systems that constrain employee behaviors to assure ethics and law compliance, and are generally made up of elements such as ethics codes, ethics committees, information systems, training programs, and disciplinary processes (Weaver et al., 1999). These elements have different orientations. For example, some elements focus on coercive control, e.g., punishment, system monitoring, and rules, while some elements focus on the support for employees’ development of attitudes and commitment, e.g., ethical training and ethics codes (Weaver et al., 1999; Michaelson and Tosti-Kharas, 2019; Teresi et al., 2019; Hauser, 2020; Vveinhardt, 2022). In the framework of Merchant and Van der Stede (2012), the organization control system is categorized into personnel and culture control practices, action control practices, and result control practices. Personnel and culture control practices refer to socializing personnel’s values and beliefs to match the organizations’ preference by training them and giving them guidelines and resources that facilitate tasks to be conducted properly, e.g., ethical training and ethical committee development (Kaptein, 2009; Merchant and Van der Stede, 2012; Murcio and Scalzo, 2021). Action control practices refer to managing the specific routine and some actions by the process control approach and a series of systems, e.g., process control and certain auditing systems (Van Hooft et al., 2005; Kaptein, 2009; Merchant and Van der Stede, 2012; Endenich and Trapp, 2020). Result control practices refer to managing behaviors by the provision of reward incentives and punishments according to realized results and specific practices, e.g., reward and punishment approaches (Kaptein, 2009; Merchant and Van der Stede, 2012; Jannat et al., 2022; Shang and Yang, 2022). The literature on the practices of ethics programs has proposed some common practices and their categorizations, but has not been definitive about the major practices within each category of ethics programs. Thus, identification and classification of ethics practices will contribute new and useful insights to the literature.

### Service employee ethical behaviors and ethics programs

Employee ethical behavior refer to individuals’ behaviors that comply with widely accepted moral norms at the workplace, e.g., helping co-workers and having concern for customer interest (Moore et al., 2012). The literature indicates that employee ethical behaviors can be encouraged through both informal (e.g., cultivating ethical culture and employee ethical cognition) and formal ways (ethical training, ethics codes, and evaluation system) (Kaptein, 2009; Moore et al., 2012; Tian and Peterson, 2016; Qing et al., 2019). Studies focusing on the former mainly apply theories such as Theory of Reasoned Action (TRA) (Fishbein and Ajzen, 1975), Theory of Planned Behavior (TPB) (Ajzen, 1991; Dewberry and Jackson, 2018), Four-component Analysis (Rest, 1983), and Cognitive Moral Development Theory (Treviño et al., 2006). They have already identified a range of important factors, e.g., ethical culture and moral identity (Treviño et al., 2006; Moore et al., 2012; Watley, 2014; Metwally et al., 2019; Cabana and Kaptein, 2021) and tended to focus on using cognitive approaches to facilitate individuals’ moral development to determine how to judge, proceed, and solve ethical dilemmas. Informal ways (e.g., culture) take a long time to influence employee behaviors (Moore et al., 2012; Warren et al., 2014). In the psychological filed, some literature also proposes the importance of ethical leadership and ethical training to enhance business ethics (e.g., Murcio and Scalzo, 2021; Wang et al., 2021). Thus, many organizations also choose to adopt formal ethics programs (e.g., ethical education and training) (Izzo, 2000; Remišová et al., 2019) to enhance employees’ behaviors, leading to the proposition that ethics programs are indispensable to organizations in developing employee ethics. However, many empirical studies focus on the effectiveness of one element in ethics programs (e.g., ethics codes) and have obtained only limited insights into how to influence employee behaviors. For example, ethics codes can clearly reflect employee ethical requirements and offer guidelines for employee ethical decision-making, while the debate on whether they can reduce the unethical behaviors continues to exist. Indeed, the literature on employee ethical behaviors is still very limited in its understanding of the control practices within these ethics programs that are effective in enhancing employee ethical behaviors.

## Research methods

### Research design

Firstly, based on Merchant and Van der Stede (2012)’s framework and some literature, the practices within ethics programs can be categorized into personnel and culture control practices (e.g., ethical training, ethics codes, and ethical committee development), action control practices (e.g., reward schemes and punishment approaches). Among such many ethical practices, which practices have commonly introduced in the professional industry and which ones are the potentially effective practices that can indeed enhance employee ethical behaviors in the context of professional service, and which are their relevant contextual factors need be examined. Thus, we set Study 1 as exploratory approach research methods to highlight which practices within ethics programs can take effect role in reducing unethical behaviors and which key contextual characteristics have some leverage roles. The samples can be selected from five main Chinese banking organizations based on the rank of revenue and profitability published by Fortune magazine of year 2021. And then, we set Study 2 to seek to develop a deeper understanding of the relationships among identified key practices within ethical program, employee ethical behaviors, and contextual factors in randomly selected 146 frontline banking teams in five main Chinese banking organizations. Also, the use of a survey in Study 2 helps addressing the potential problem of limited generalizability of Study 1.

### Study 1

While professional service companies (e.g., financial institutes) are critically important for almost all economies and such organizations always have varying types of ethics programs in place, the literature on ethics programs has offered no systematic framework guiding ethics program decisions in professional service companies (Weaver et al., 1999). No wonder many professional organizations may find it difficult to identify which ethics programs are effective in the specific ethical problems they are facing and what are the main contextual factors influencing the relevant implementations. This suggests the need for inductive research to help answer these questions.

#### Case selection and research setting

The research setting focuses on the Chinese financial banking industry. There are three reasons. Firstly, financial banking industry is a typical service industry and involves significant characteristics of service operations with customized service and no inventory, flexible and uncertain processes, and co-production with customers (Bowen and Ford, 2002; Safizadeh et al., 2004). Secondly, most ethical scandals publicized by a wide variety of media are highly related to the employees of financial industry; it is partly attributed to their customized professional service (Fusaro and Miller, 2002; Zhou, 2006). Lastly, The Peoples’ Bank of China (2002), China’s major policy bank, regularly issue business codes and guidelines with banks documents to control professional behaviors. Thus, the financial banking industry represents an appropriate context to explore our study.

Based on the rank of revenue and profitability published by *Fortune* magazine of year 2021, this paper chooses five Chinese banking organizations, i.e., A, B, C, D, and E, which are listed under Fortune global top 500 business. The financial revenue of these five banks is at least 70,000 million USD per year and their regulations and codes of operational procedures are issued and improved every year. So from these banks, more information about these problems can be obtained, e.g., which practices have been implemented in ethics programs from a professional service context; which practices are effective to strengthen employee ethical behaviors; and which contextual factors mainly influence their effectiveness. Considering that professional service sectors are increasingly relying on frontline teams to generate sales opportunities and implement the various kinds of tasks needed (Mills et al., 1983; Schultz et al., 1999), this paper randomly chose ten frontline teams with high comprehensive performance rank involving one or two team leaders and several team members from the five sample banks. Table 1 shows the position, the number of informants in this position, the times of interview and the stage of data collection. Taking into account the cognitive bias between employee and leader, we identified two excellent teams per sample bank, i.e., ranked as top two, with superior comprehensive performance including ethical aspects. The teams were nominated by managers from the respective headquarters who asked their teams to inform us on the effectiveness of practices in ethics programs.

**TABLE 1:** Informant information.

Corporate name	Position	Informants no.	Interviews no.	Stage of data collection
A bank (two teams)	Vice banker of bank branch	1	2	3
	Frontline employees	3	1	2
	Directors of credit card	1	1	2
	Frontline employees	3	1	2
B bank (two teams)	Customer manager	1	2	3
	Frontline employees	4	1	2
	Banker of bank branch	1	1	2
	Front line employees	3	1	2
C bank (two teams)	Manager of bank branch	1	2	3
	Frontline employee	2	1	2
	Customer manager	1	1	3
	Frontline employee	3	1	2
D bank (two teams)	Banker of branch	1	2	3
	Frontline employee	3	2	3
	Customer manager	1	1	2
	Frontline employee	4	1	2
E bank (two teams)	Customer manager	1	1	2
	Frontline employees	3	2	3
	Customer manager	1	1	2
	Frontline employees	2	1	2
Total no.		40	26	

#### Data collection

The data for this study was collected from several sources. First, we obtained qualitative and primary data by interviewing ten leaders and 30 frontline employees selected from five major banks, i.e., A, B, C, D, and E. All the interviews were semi-structured and face-to-face, and each of them lasted about 3 h. Quotes from the interviews with Chinese informants were recorded and then translated into English for analysis. Example questions include “What are the constituent practices of the ethics program?”, “What is the rationale in formulating the program?”, “Which practices are the most effective?”, “What contextual factors influence the effectiveness of the program?” Second, we obtained information on how the sample banks decided their business regulations and how they solved some common ethics issues by examining their daily operation routines records, internal control documents, and some related archival data (e.g., violation investigation files). Finally, after conducting the preliminary analysis, we clarified some inconsistencies and ambiguities with the corresponding respondents via telephone and e-mail.

#### Data analysis

We adopted data triangulation (Yin, 2003) to collect and compare data from different respondents (team leaders and team members) to enhance the validity and reliability of the case study. At the same time, following Yin (2003), we conducted between-method triangulation based on the multiple resources used (interview, communication via email, archival data and internal corporate documentation) to capture exploratory phenomena (Langley, 1999). We analyzed and compared the data by using cross-case analyses and generalized the results into findings (Caniato et al., 2012). Specifically, we used open coding to analyze the quantitative data. The steps involved identifying the similarities or common characteristics of the data and texts by reading, observing, and comparing the data, and grouping the common characteristics under same group. We labeled the concepts and categories based on the dimensions of control practices described in Merchant and Van der Stede (2012)’s framework and properties of data, marked the text, and set proper codes as identification tags. See Tables 2–4 for the findings.

**TABLE 2:** Content analysis of control practices relevant to ethical behavior in the professional service context.

Company	Representative quotations	Open coding for practices
Bank A	… *There are four systems to control employee behaviors including behavior-based system, supervision system, education and training, and punishment and reward approach*… …*Behavior-based system mainly use formal guidelines and codes to control employee behaviors. Supervision system uses monitoring technologies*… *the types of training are various such as on-line training, on-site training and one employee-one supervisor training*…*punishment and reward approach focus on implementing the reward and punishment on some ethical and unethical behaviors.*	(1) Formal guidelines for operational routine. (2) Monitoring technologies. (3) Training and education. (4) Punishment and reward approach.
Bank B	… *Our organization has some ethics programs such as ethics codes, daily routine policies, monitoring system and ethical training. The monitoring system includes workmate co-assessment and whistle-blowing. We adopt the approach of the case education as ethical training*… … *Organization has decision-making risk control mainly for managers*…	(1) Ethics codes. (2) Daily routine policies. (3) Workmate co-assessment system. (4) Whistle blowing. (5) Case education. (6) Decision-making risk control.
Bank C	… *Our organization developed ethics codes and implemented the behavior control for employees, for example giving some guidelines for their tasks or cultivating their risk recognition*… *We also use some customer evaluation system to monitor employee behaviors*…	(1) Ethics codes. (2) Formal guidelines for tasks. (3) Risk recognition. (4) Customer evaluation system.
Bank D	… *There are several systems to reduce unethical behaviors. Behavior control system comprising some guidelines and the methods of risk aversion, training system comprised of ethical case education, monitoring system comprising information system statistical analysis and customer evaluation*… *We use the reward approach to encourage ethical behaviors.*	(1) Formal guidelines for routines. (2) Risk aversion. (3) Ethical case education. (4) Information system. (5) Reward approach.
Bank E	…*Generally, we have ethics codes and policies on daily tasks to constrain employee behaviors*… *we also adopt the risk control for managers and employees in service decision-making*…*we also set the reporting system to report the unethical behaviors*…	(1) Ethics codes. (2) Policy relevant with behaviors. (3) Risk attitude control. (4) Reporting system.

**TABLE 3:** Content analysis of the effectiveness of the concluded practices.

Type of control	Relevant practice	Representative quotations	Open coding	Effectiveness
Culture/personnel control	Ethics codes	… *Most ethics practices are too general to guild ethical dilemma*…*;*… *we seem to find it difficult to identify specific ethics guidelines telling us how to act*…, …*we couldn’t decide on how to implement them to form culture or climate*…	(1) Too general. (2) Difficult to find/implement. (3) Outdated.	Limited
	Case education and training	… *These training and education initiatives are effective in terms of proficiency of task operations but have no significant effect on ethics in the service. Mostly, we meet the exam requirements rather than alter our behaviors*…	(1) No significant effect. (2) Just meet the requirements of examination/certification.	Limited
Action control	Guidelines for daily tasks	… *I think business codes and control mechanisms are effective, because they are more specific and involve how to implement them in our specific operational routine activities*… *I think control mechanisms involving operational routines relevant with daily tasks are effective*…	(1) More specific. (2) Effectively. control behaviors	Effective
	Monitoring technology/information systems	… *It is difficult to monitor employee behaviors because their service operation is the process of lip service*…*Intangible service seems too difficult to be monitored by some technology*… … *Information systems can reflect some mistakes in our operational behaviors, and thus is somewhat useful to enhance our ethical behaviors*… *Some statistical analysis can constrict behaviors*…	(1) Difficult to monitor. (2) Lip service. (3) Intangible service. (4) Statistical analysis. (5) Reflecting employee behaviors.	Biased effective
	Workmate co-assessment system	… *Due to close relationships with workmates, they hardly do the actual assessment*… *it’s a just superficial task*… *they are more willing to keep the consistence with his workmate*…*but sometimes this practice can get accurate evaluation.*	(1) Hardly do the actual assessment. (2) A superficial form. (3) Be consistent with their workmates. (4) Sometimes getting accurate evaluation.	Biased effective
	Whistle-blowing	… *Close relationships among workmates makes employee unwilling to whistle-blowing*… *For whistle-blowing system, we perceive that it has no effect, because we have good relationship with our workmate and are not willing to see the punishment they receive., sometimes we may be afraid of getting marginalized by other co-workers or team members, because we didn’t know the consequences of whistle-blowing.*	(1) Good/close relationship with workmates. (2) Afraid of being marginalized. (3) Hardly predicting the consequences of whistle-blowing.	Limited
	Risk aversion	… *I think our risk-related decision-making for managers is very effective*… *Risk control can help employees to reduce the risky decisions*…	Reduce risky decisions	Effective
	Customer evaluation system	… *Customers participating in some assessment can facilitate employees to implement ethical behaviors*…	Encourage employees’ ethical behaviors	Effective
Result control	Reward approach	*The reward related to ethics behaviors is too subjective and we seem to have no idea about its metric and how to implement them, even if we want to implement them.*	(1) Too subjective. (2) Difficult to implement. (3) Lack of metrics.	Limited
	Punishment approach	*As for punishment regulations, managers generally give warnings rather than implement the regulations, because they prefer to give us correction opportunities and to avoid disruptions in the routine operations.*	Only warning.	Limited

**TABLE 4:** Perceived main contextual factors impacting the effectiveness of control practices.

Main representative quotations	Open coding
… *Sometimes there is some role conflict between corporate policies and customer expectation so we find it hard to follow control practices to deal with these dilemmas. When these dilemmas occur, most of the team members (or managers) firstly consider self–interests*… *we at times find our team goals (mostly, about efficiency and accuracy) and our individual goals (mostly, sales quota) actually are contradicting*…	(1) Role conflict. (2) Goal conflict.
*In most situations, we have some work stress and burden, and are required to complete the work as soon as possible. So we are concerned less about cost, reputation problems and long-term profit, and just expect to get normal wages and benefit monthly. We perceive that it’s not necessary to follow these ethical control practices that can serve the management (and some front-line employees)*	Work stress and burden
*There is some disorder and unfair competition among banks, so every bank has to set a high or even very difficult to achieve task goal to maintain existing status. Every employee also receives rigid assignments that are difficult to complete. If they could not complete them within the specified time requirement, they will get some punishment, such as wage reduction, demotion. Time pressure and workload make employee seek more shortcuts (e.g., misrepresenting product information to customers) rather than following the control guidelines (some managers).*	(1) Time pressure and workload. (2) Difficult to achieve task goals.
*We are tired every day and have no time to consider how to follow ethical control programs. We just focus on completing our tasks, in order not to jeopardize our wages or get demoted. Even if we introduced all aspects of some products, the banks wouldn’t give us more money. Most ethical control programs protect management to avoid getting blamed (some front-line employees).*	Tired
*Indeed, our members don’t know clearly how to use control practices to achieve their sales quota. We focus primarily on observable results and get them in the simplest way (frontline employees).*	The paucity of information and guidelines

#### Findings

##### Main practices of ethics programs in professional organizations

We coded the main practices of ethics programs from our five cases in Table 2. They could be coded as formal guidelines related to operational routines, monitoring technologies, education and training, punishment and reward approach, ethics codes, workmate co-assessment, whistle-blowing, risk aversion, information system, customer evaluation system, and reporting system. Following the framework of control systems (Merchant and Van der Stede, 2012), these were categorized into three different kinds of control systems (see Table 3): cultural/personnel control systems includes ethics codes, education, and training. Action control systems include guidelines for daily tasks, monitoring technologies/information system, workmate co-assessment, whistle-blowing, risk aversion, and customer evaluation system. Result control systems include reward and punishment approaches. These findings enrich the literature on ethics programs that include three or four dimensions. In addition, based on these practices, we further identified the types of programs that were most effective for enhancing ethical behaviors from the coding results.

##### Effective control practices of ethics programs

Table 3 lists the specific practices classified into cultural/personnel control system that have limited effectiveness. For example, ethics codes are too general and outdated to be able to guide their behaviors. Further, training and education is generally so boring for employees that its effectiveness can be poor. Also, result control system (e.g., reward and punishment approach) have limited effectiveness, since the metric of reward and punishment, i.e., behavior-oriented outcomes, are difficult to be measured accurately. However, the formal guidelines for daily routines, risk aversion and certain information measures in the action control system shown in Table 3 are effective in enhancing employee ethical behaviors. According to the literature on behavior control and agency theory (Smith and Karwan, 2010; Goodale et al., 2011), the three effective practices from the case study are consistent with the dimensions of behavior control, i.e., process control formality, risk control, and information system intensity. Specifically, formal guidelines for service processes aimed at reducing uncertainties arising during performing tasks can be defined as process control formality. The adoption of risk aversion and the pursuit of stable profits in products and services can be conceptualized as risk control. More and more organizations will make a trade-off between performance improvement and risk aversion. Considering risk control in the process of product and service innovation is necessary (Shin et al., 2022). Finally, the degree of accessibility, the frequency, and richness of the information system embedded in the service process can be conceptualized as information system intensity.

##### Main contextual factors highly relevant with the effectiveness of these practices

Table 4 indicates that the main contextual factors of influencing ethics practices are task conflict, goal conflict work stress, time pressure and workload, difficult achievable task goals and role ambiguity. Based on role stress theory, we can say that these factors can reflect the employee suffering from role stress. Job stress is an important signal that frontline employees are likely to experience serious burnout, which is a kind of role stress (Choi et al., 2019). Also, time pressure, workload, and difficult achievable task goals can be included in role overload. Task conflict and intra-group conflict can be perceived as role conflict. Paucity of information and guidelines in the work environment are defined as role ambiguity (Nordenmark, 2004). Our findings suggest that role stress in professional organizations is an important element of job climate. In addition, from the quotations, we find that service professionals suffering from role stress climate perceive that behavior control practices are not beneficial to their tasks (e.g., increasing sales quotas) but just help the management to avoid problems or blame from the public. Because of such negative attitudes toward control practices, we predict that frontline professionals may not genuinely follow the control practices while completing their daily tasks. Role stress climate may be an important factor impacting the effectiveness of behavior control practices.

#### Discussion

The findings from Study 1 demonstrate that our sample organizations had implemented a variety of ethics programs including cultural and personnel control systems (e.g., ethics codes and employee training and education), action control systems (e.g., process control and risk aversion) and result control systems (reward and punishment approaches) to enhance employee ethical behaviors. Also, managers and employees had perceived that behavior control practices (e.g., process control formality, risk control, and information system monitoring) were effective in solving agency problems in frontline contexts. Thus, it can be said that agency problems (e.g., employee engaging in certain behaviors in response to personal interests at the expense of corporate reputation and profitability) represent a major factor influencing the professionals’ ethical behaviors. In addition, we find that the main professional contextual factors relate to role stress, i.e., role ambiguity, role conflict, and role overload, which may impede employees from implementing the practices effectively. These results suggest that there should be some relationships among behavior control practices, employee ethical behaviors, and role stress climate. However, since our qualitative results are based on ten teams from five banks, they cannot be generalized to teams from different professional service organizations. Also, the qualitative findings do not give specific insights about the relationships among behavior control practices, ethical behaviors, and role climate. To address these issues, we conducted a quantitative study.

### Study 2

**FIGURE 1:**
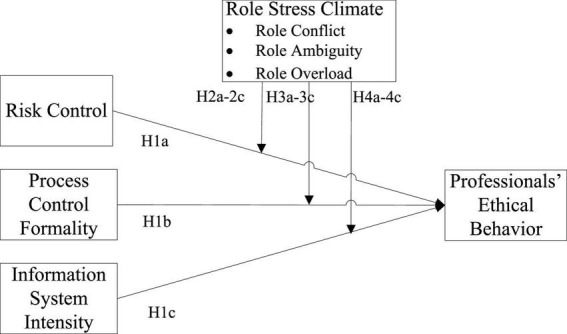
The research model.

Bases on Study 1, the objectives of Study 2 were to examine (1) whether behavior control practices, i.e., risk control, control process formality, and information system intensity, are positively associated with professionals’ ethical behaviors and (2) whether role stress climate weakens the effectiveness of behavior control practices in ensuring professionals’ ethical behaviors. Because behavior control and role stress climate are highly relevant to agency theory and role stress theory. Study 2 first integrated the two theories with the ethical literature to develop our hypotheses (see Figure 1) and then collected data from 146 frontline professional teams working from 5 major China’s banking industry conducted by case study and used them as analysis units to test our hypotheses. The findings of Study 2 complement the qualitative analysis and offer the precise relationships among control practices, ethical behaviors, and contextual factors.

#### Hypothesis development

##### Relationship between behavior control practices and service professionals’ ethical behavior

From a review of the literature on agency theory, risk control, process control formality, and information system intensity are included in behavior control practices with the objective to alleviate the agency problem, i.e., moral hazard (Morgan et al., 2007; Goodale et al., 2008). Indeed, in the context of professional organizations (e.g., high levels of discretion and autonomy, employee specialized knowledge, and a view to enhancing selling performance) (Goodale et al., 2008; Von Nordenflycht, 2010; Lewis and Brown, 2012), the question of the moral hazard associated with ethical performance frequently arises (e.g., employees exaggerate the attributes of products to achieve sales due to information asymmetry). Behavior control practices from the perspectives of organizational climate, task structure, information system, and operational procedures are usually established to restrict employee behaviors and offer clear behavioral guidelines and task requirements regarding how frontline professionals serve customers and how they can avoid risky behaviors (Goodale et al., 2011), which can lower the frontline service professionals’ discretion levels and reduce opportunities for risky decision-making, thereby assuring the organization’s reputation and long-term profitability.

Specifically, risk control and process control formality involve setting up organizational boundaries related to acceptable behavior definitions, performance-related expectations, codes to be met, and regulations to be followed so as to prevent employees’ work deviations from what is expected from different perspectives, mainly attitude and process (Das and Joshi, 2007). In circumstances with clear guidelines and restrictions, professionals are usually inclined to carry out activities and behave ethically in a manner consistent with their organization’s regulations and standards (e.g., ethics codes), thereby solving the agency problem of moral hazards and enhancing professionals’ ethical behaviors. Complicated information systems can track many operational problems such as monitoring employees’ total service process, and obtaining customer feedback effectively. This can help operational managers glean more information about employee behaviors, thereby encouraging employees to follow ethical guidelines and reduce risky decision-making on their part. Thus, considering the complexity of service processes in professional organizations, behavior control practices embracing risk control, process control formality, and information system intensity can become effective means to improve employee ethical performance. From this perspective, the study proposes the following hypothesis:

H1: Behavior control practices with respect to (a) risk control, (b) process control formality, and (c) information system intensity in professional organizations can effectively enhance professionals’ ethical behaviors.

##### Moderating effect of role stress climate

Our case studies showed us that role stress climate has become an important element of job climate in professional organizations. Professionals with role conflicts between activities relevant with performance assessment requirements (e.g., sales quota) and activities with organization reputation (e.g., ethically serving customers), may not comply with the risk control practices to cautiously pursue long-term organizational reputation, but give priority to activities with short-term performance requirements. Also, they may believe that step-by-step structured practices have been developed to mitigate operational risks and increase organizational brand value rather than protect them and meet their personal performance requirements. This leads to certain negative attitudes while following process procedures. When frontline teams with role overload feel detached from their jobs and become unwilling to respond to customers (Nordenmark, 2004; Yagil et al., 2008), they are unlikely to adopt the cautious attitude needed to deal with relevant tasks and become cynical about cumbersome and repetitive step-by-step procedures and processes, i.e., process behavior formality. When professionals with role ambiguity are lack of enough information and guidelines, it is difficult to require them to follow risk control practices and methods to engage in proper decision-making. In addition, although there may be information systems tracking and monitoring employee behaviors, professionals suffering from role stress climate use their limited time and capacity to complete only top-priority tasks to stay on in their positions. Behaving ethically is highly relevant from the viewpoint of the long-term profitability and reputation of the organization rather than merely from the viewpoint of employees’ personal performance assessment. This limits the ability of information system control and monitoring aimed at encouraging ethical behaviors. For example, in China’s banking industry, on top of the frontline professionals’ regular service activities, employees have to carry out more activities such as reading legal statements to customers, conducting customer screening surveys, and completing forms for documentation purposes. Indeed, some studies demonstrate that ethical behaviors or proactive behaviors have close linkage with role stress (e.g., Strauss et al., 2017). Thus, when frontline professionals experience role stress, risk control, process control formality and information system control will play a curtailed role in improving their ethical performance.

H2: The role stress climate arising from (a) role conflict, (b) role ambiguity, and (c) role overload of frontline professionals in organizations has a negative impact on the relationship between risk control and their ethical behaviors.

H3: The role stress climate arising from (a) role conflict, (b) role ambiguity, and (c) role overload of frontline professionals in organizations has a negative impact on the relationship between process control formality and their ethical behaviors.

H4: The role stress climate arising from (a) role conflict, (b) role ambiguity, and (c) role overload of frontline professionals in organizations has a negative impact on the relationship between information system intensity and their ethical behaviors.

#### Research methods

##### Research samples and procedures

In Study 2, we collected the sample from nine major banks located in seven economically developed cities of China. The banks included five state-owned commercial banks, three second-tier nationwide commercial banks, and one city-based commercial bank. To develop the sampling frame, we first examined the Internet web-pages of the target banks to search for the contact information of their frontline departments. By making calls to those departments, we identified the team leaders and explained to them the objective of our study. In the event, we came up with a sampling frame comprising 400 frontline teams among the target banks. We also adopted a multiple-informant method to collect data from one leader and three randomly-selected members in each team.

Since employee ethical behavior is a new construct, we paid extra attention to gather insights from the case study to identify the relevant items and improving their readability. Also, the findings from our case studies indicate that team leaders should be suitable to fill in constructs on the frontline employees’ ethical behaviors and the teams’ adoption of control mechanisms, whereas the team members are likely to be more knowledgeable regarding the stress they experience at the workplace. Thus, the leader questionnaire included the constructs of employee ethical behavior and those regarding control practices, whereas the member questionnaire included the constructs of role stress. To enhance the response rate in the survey, we issued three rounds of reminders. At the end, we received usable data from 146 teams (each team has one leader questionnaire and three member questionnaires), resulting in a response rate of 36.5%.

##### Measures

This study used a multi-item, seven-point Likert scale anchored at 1 = “totally disagree” and 7 = “totally agree” to measure all the constructs and discuss the construct items in the following.

*Risk control* refers to frontline service professionals’ risk-taking attitudes. We used four items adapted from Das and Joshi (2007) and Goodale et al. (2011). Sample items included “our team adopts a cautious posture in order to minimize the probability of making costly decisions in the operation process” and “explores some knowledge from the internal and external environments via cautious and incremental behavior” (α = 0.840).

*Process control formality* refers to the extent to which frontline service professionals need to follow formal and systematic procedures while carrying out their service processes. We used the four items developed by Das and Joshi (2007) and Goodale et al. (2011). Sample items included “our team follows formally laid-down procedures” and “…gets in line and adheres closely to formal job descriptions” (α = 0.915).

*Information system intensity* refers to the frequency and richness of system monitoring, evaluating, and dealing with problems in service processes. This study adopted the five items developed by Smith and Karwan (2010). “Sample items included information system supports professional operation activities” and “A database is maintained for tracking and monitoring mistakes and failed performance” (α = 0.937).

*Role stress climate* refers to respondents’ shared perceptions of role stress in their work climate, including three main and different stressors, namely, role ambiguity, role conflict, and role overload. This study adopted these items from Rizzo et al. (1970) and Beehr et al. (1976). Sample items of role conflict [α = 0.932, ICC (1) = 0.575, ICC (2) = 0.803], role overload [α = 0.936, ICC (1) = 0.654, ICC (2) = 0.850] and role ambiguity [α = 0.960, ICC (1) = 0.343, ICC (2) = 0.610] included “We receive incompatible requests from our customers and our organization,” “too much work for one person to do” and “no clear, planned goals and objectives for our job,” respectively.

*Professional ethical behavior* refers to the extent to which employees are able to (1) perform their activities in accordance with ethical standards, (2) cope with ethical dilemmas to maintain ethical standards, and (3) propose approaches to resolve future ethics-related problems. We developed six items (α = 0.967) by adapting the concepts of work role behaviors (Griffin et al., 2007) and performance appraisal (Behrman and Perreault, 1982). Sample items included “our team concerns customer benefits when conduct codes are not adequate” and “…contributes during team discussions for improving ethics in the team.”

*Control variables* consist of the informant’s age and education level, functional department, the position of the team leader (e.g., teller, retail and integrated service manager, financial manager, account manager, and credit manager), and the duration for which the informant has worked on the team. According to the literature, employee age, education, working tenure, bank’s department and the position of the leader may influence professional ethical behavior (Birkelund, 2000; Werner et al., 2005; Lin and Wei, 2006). By removing the effects of these control variables in our analyses, we were able to test our hypotheses more accurately.

##### Reliability and validity

Since the unit of analysis of this study is a frontline professional team, we first employed the intra-class correlation coefficient (ICC) statistics ICC (1) and ICC (2) to assess the viability of aggregating individual-level data from three team members to the team level (James, 1998). The results shown in the Measures part indicate that all the individual-level data can be aggregated to form team-level data based on James (1982)’s suggestion that the value of ICC (1) should be above 0.12 and Boyer and Verma (2000)’s suggestion that the generally acceptable level of ICC (2) in the field of OM should be above 0.60. Regarding reliability, the lowest α-value is 0.84 (see the Measures part), which is acceptable (George and Mallery, 2003). To assess its general validity, we compared the major model’s fit results with those from the competing models. The resulting fit indices indicated that the major model had a better fit than the competing models and its results for the fit indices were above the suggested threshold values (Byrne, 2013). In addition, the results on composite reliability (CR) and average variance extracted (AVE) ranging from 0.824 to 0.97 and from 0.543 to 0.900, respectively, revealed adequate discriminant validity and convergent validity in the constructs examined. Finally, we addressed the potential impact of common method bias by employing the two approaches recommended by Podsakoff et al. (2003). We conducted two statistical tests. The first was Harman’s one-factor test where the results indicated that only 42.06% of the common method variance could be explained by this major factor. Thus, we obtained adequate evidence that common-method bias was unlikely to be a significant concern with our data. Table 5 displays the means, standard deviations, and correlations associated with all the variables.

**TABLE 5:** Means, standard deviations, and correlations among the study variables.

Variables	Mean	S.d.	1	2	3	4	5	6	7	8	9	10	11	12
(1) Education level	2.11	0.37												
(2) Age	1.36	0.34	–0.041											
(3) Post	2.04	0.99	−0.194*	0.069										
(4) Department size	2.88	0.80	–0.107	0.193*	0.049									
(5) Working time	3.59	0.53	–0.127	0.170*	–0.126	0.263**								
(6) Risk control	5.44	0.87	–0.096	0.058	0.111	0.099	0.182*							
(7) Process control formality	5.68	1.09	−0.169*	0.092	0.256**	0.230**	0.141	0.444**						
(8) Information system intensity	5.41	1.02	−0.216**	0.004	0.063	0.067	0.187*	0.355**	0.406**					
(9) Role conflict	4.03	1.39	0.096	–0.018	–0.140	0.018	–0.151	−0.354**	−0.224**	−0.291**				
(10) Role ambiguity	2.37	0.88	–0.046	–0.121	0.036	–0.062	–0.162	−0.269**	–0.129	−0.231**	0.474**			
(11) Role overload	3.88	1.55	0.168*	–0.072	−0.280**	–0.024	−0.208*	−0.242**	−0.250**	−0.233**	0.702**	0.403**		
(12) Employee ethical behavior	5.58	1.16	–0.062	0.165*	–0.016	0.081	0.271**	0.338**	0.377**	0.236**	−0.212*	−0.321**	–0.160	1

*p < 0.05, **p < 0.01.

##### Hypothesis test

We employed hierarchical regression analysis to test our hypotheses. Table 6 shows the regression results where the *F* values in all the models are highly significant (*p* < 0.01). In Models 2, 3, and 4 shown in Table 6, the results indicate that the control mechanisms, i.e., risk control, process control formality, and information system intensity, are positively and significantly associated with employee ethical performance (β = 0.300, *p* < 0.01; β = 0.379, *p* < 0.01; β = 0.199, *p* < 0.05), thus supporting Hypotheses 1a–1c.

**TABLE 6:** Hierarchical regression results for Hypotheses 1–3.

Dependent variable Operational performance	Model 1	Model 2	Model 3	Model 4	Model 5	Model 6	Model 7
**Step 1: Control variables**							
Education	–0.026	–0.012	0.008	0.011	0.031	0.003	0.019
Age	0.123	0.120*	0.116*	0.131*	0.061	0.105	0.101
Post	0.003	–0.034	–0.090	–0.008	0.022	–0.058	0.020
Department size	–0.011	–0.023	–0.078	–0.012	0.020	–0.067	–0.007
Working time	0.250**	0.196*	0.208*	0.215*	0.181*	0.181*	0.189*
**Step 2: Independent variables**							
Risk control		0.300**			0.279**		
Process control formality			0.379**			0.401**	
Information system intensity				0.199*			0.134
Moderating variables							
Role conflict					0.039	–0.053	0.031
Role ambiguity					−0.321**	−0.246**	−0.276**
Role overload					0.041	0.102	0.081
**Step 3: Two-way moderator effect**							
Risk control * Role conflict					0.058		
Risk control * Role ambiguity					−0.357**		
Risk control * Role overload					–0.013		
Process control formality * Role conflict						0.112	
Process control formality * Role ambiguity						0.009	
Process control formality * Role overload						−0.191*	
Information system intensity * Role conflict							0.074
Information system intensity * Role ambiguity							−0.172^+^
Information system intensity * Role overload							–0.067
*F*	2.72*	4.87**	6.26**	3.31**	5.10**	4.62**	2.97**
*R* ^2^	0.089	0.174	0.213	0.125	0.315	0.294	0.211
Δ*R*^2^	Nil	0.085	0.039	–0.088	0.190	–0.021	–0.083

^+^*p* < 0.1, *p < 0.05, **p < 0.01.

**FIGURE 2:**
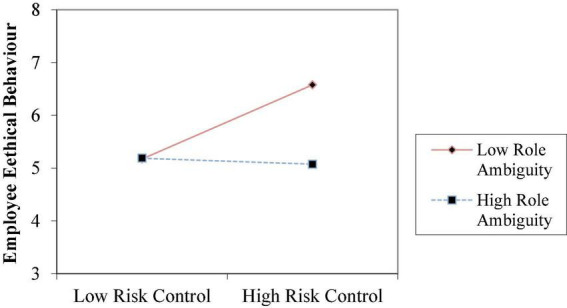
The moderating effect of role ambiguity on the relationship between risk control and employee ethical behaviors.

**FIGURE 3:**
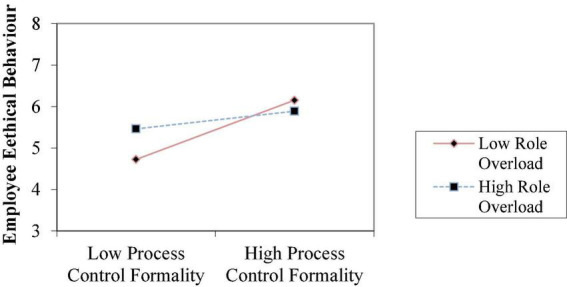
The moderating effect of role overload on the relationship between process control formality and employee ethical behaviors.

**FIGURE 4:**
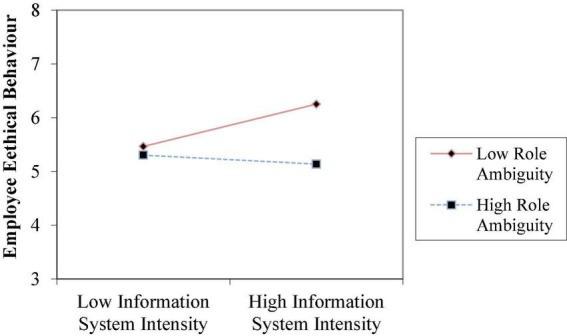
The moderating effect of role ambiguity on the relationship between information system intensity and employee ethical behaviors.

To test Hypotheses 2a–2c, 3a–3c, and 4a–4c, we estimated the interaction effects in the regression model using the cross-product terms and entering each cross-product term separately to avoid multicollinearity (Gopal et al., 2012). As for Model 5, the results pertaining to the significant coefficient of the interaction term (β = −0.357, *p* < 0.01) and the observed significant increment in *R*^2^ in Models 5 over Model 2 (0.141) supported Hypothesis 2b, indicating that role ambiguity can indeed weaken the association between risk control and professional ethical behavior. Figure 2 shows when employees suffer more role ambiguity, risk control takes less effect on their ethical behaviors. In Model 6, the results pertaining to the two-way interaction coefficient (β = −0.191, *p* < 0.05) are significant and negative. Model 6 accounts for a large amount of variance in the dependent variable (*R*^2^ = 0.294), and is well above and beyond the amount explained by their corresponding main effect in Model 3 (*R*^2^ = 0.213). These evidences supported Hypothesis 3c that role overload has a negative impact on the relationship between process control formality and their ethical behavior. Figure 3 shows when employees suffer more role overload, process control formality takes less role in improving their ethical behaviors. In Model 7, the results regarding the significant coefficient of the interaction term (β = −0.172, *p* < 0.1) and the significant rise in *R*^2^ in Model 12 over Model 4 (0.086) support Hypothesis 4b, indicating that role ambiguity can weaken the association between information system intensity and professional ethical behaviors. Figure 4 shows when employees suffer more role ambiguity, information system intensity have less effect on their ethical behaviors. Note that the coefficients of the interaction terms in other Models are not significant.

#### Discussion

Study 2 has examined the effectiveness of behavior control practices on employee ethical behaviors under the major context, i.e., role stress climate. Its findings suggest that behavior control practices can indeed improve professional ethical behavior, which is consistent with the finding of Study 1. Good behavior control practices offer clear behavioral guidelines on service operations. Specifically, low control behavior often leads to antisocial behavior (Tehrani and Yamini, 2020). For example, lowering professionals’ discretion levels will affect their attitudes toward goal conflicts and risky behaviors, thereby reducing risky decision-making on their part, as well as encouraging them to be more concerned about customer benefits and long-term organizational profitability. Also, our results from Study 2 indicate that role ambiguity has a negative influence on the relationship between risk control and ethical behaviors, and between information system intensity and ethical behaviors. A climate charged with role ambiguity often lacks sufficient information so that professionals cannot clearly learn how to proceed with their tasks efficiently while striving to meet performance expectations from their organization (Singh, 1998). Under such circumstances, risk control and information system intensity play limited roles in restricting their risky decision-making, which encourages them to behave unethically. In addition, our findings indicate that role overload can moderate the relationship between process control formality and professionals’ ethical behaviors negatively. Besides, our findings concerning the moderating effects of role overload and role conflict on the relationship between risk control and a professional’s ethical behaviors and the relationship between information system intensity and professional’s ethical behaviors are not significant.

## Conclusion

In this manuscript, to effectively enhance marketing practitioners’ ethical behaviors and the pertinent contextual factors that have a bearing on the effectiveness of ethics programs, we use a multi-method methodology to conduct two studies in the Chinese banking setting. Specifically, we connect ethics programs with psychological characteristics of frontline employees, i.e., role stress, to explore how behavior control practices can take effect role in influencing employees’ ethical behaviors.

We answer the three research questions as follows: For the first research question, we investigate whether the ethics programs of service organizations are effective in enhancing employee behaviors. We find that not all ethics programs are effective in enhancing employee behaviors. Specifically, the cultural/personnel control system, training, education, and result control system have limited effectiveness. However, the formal guidelines for daily routines, risk aversion and certain information measures in the action control system are effective in enhancing employee ethical behaviors. For the second research question, we study the contextual characteristics that influence the effectiveness of the relevant practices. We find that the main contextual factors of influencing ethics practices are task conflict, goal conflict work stress, time pressure and workload, difficult achievable task goals and role ambiguity. For the third research question, we examine the specific relationship among the practices, contextual factors, and employee ethical behaviors. We show that the control mechanisms, i.e., risk control, process control formality, and information system intensity, are positively and significantly associated with employee ethical performance. Role ambiguity can indeed weaken the association between risk control and professional ethical behavior, as well as information system intensity and professional ethical behaviors. And role overload has a negative impact on the relationship between process control formality and their ethical behavior.

### General discussion

While many service organizations claim to implement several control systems as ethics programs, ethical scandals are being reported at disturbingly regular intervals. The hesitation of many organizations not to proactively engage in ethics programs is backed by some literature demonstrating that control systems seem to have no effect in reducing unethical behaviors in survey data (Mendoza, 2013). This paper has found from the case studies conducted that many practices of ethics programs implemented by service organizations have no significant effect on employee ethical behaviors, including ethical education and reward approach relevant with ethics. Based on evidence cutting across two studies, this paper has found that behavior control practices are effective in addressing employee ethical behaviors and role stress problems as main contextual factors could be relevant with their effectiveness. Study 2 had further found that the effect of behavior control practices is weakened when role stress is high.

In a professional service sector, professionals afflicted by role overload are more likely to use their professional knowledge to try out more efficient work approaches, which means that they can become cynical about specific and structured guidelines, which can slow down work process. In short, role overload can influence the effectiveness of process control formality. Since frontline employees in the professional service sector usually have certificates of training, one can assume that their knowledge intensity is high enough. However, while experiencing role overload and role conflict, such professionals can develop a cautious attitude while carrying out routine and customized tasks, and be concerned about license cancelation arising from information system monitoring and tracing. Role ambiguity and role conflict have no moderating effects on the relationship between process control formality and the ethical performance of professionals. A plausible explanation is that when an employee does not have clear tasks, goals, and requirements, process control formality can offer systematic methods to facilitate task completion and encourage behavior toward performing ethically. Professionals suffering from a climate of role conflict may continue to follow structured procedures to avoid situations that are likely to lead to work license cancelation. Further studies studying the impact of different types of role stress on the effectiveness of the behavior control mechanisms should be useful.

### Theoretical implications

The first theoretical contribution of this study relates to the use of the case study to arrive at specific behavior control practices toward improving professional ethical behaviors and further examine their effectiveness by field survey in the professional service sector. Although several cognitive and organizational practices capable of encouraging ethical behaviors have been identified in the literature and theories, ethical scandals have continued to appear in the professional service sector. This suggests that these general approaches may be playing a limited role. Indeed, from Study 1, this paper has found that behavior control practices such as risk control, process control formality, and information system intensity, i.e., various types of information systems, are effective among the ethics programs they have implemented and the main contextual factors in professional service are role stressors. Study 2 has integrated agency theory with findings from Study 1 to develop the hypotheses and reexamine the explicit relationship among behavior control practices, employee ethical behaviors and role stress climate comprised by different role stressors.

The second theoretical contribution of the study lies in the integration of role stress theory with the literature on behavior control and ethics to study the negative effect of role stress on the effectiveness of behavior control practices in improving ethical behaviors. Study 1 has identified that role stress climate is an important contextual factor highly relevant with the level of control practices implementation. Study 2 further finds that the effectiveness of behavior control practices in improving ethical behaviors is weakened under the role stress climate. These findings enrich role stress theory and behavior control practices.

The third theoretical contribution of the study is the finding that role stress climate caused by different stressors (role conflict, role ambiguity, and role overload) have differing moderating effects on the effectiveness of different behavior control practices. Integrating role stress theory with the findings of Study 1, this study identify three dimensions of role stress climate (role ambiguity, role stress, and role overload) as important contextual factors impacting control practices. Study 2 find that role ambiguity has a significant and negative influence on the effectiveness of risk control and information system intensity in improving ethical performance, while role overload has a significant and negative influence on the effectiveness of process control formality. However, more work is needed to fully clarify the influences of different types of role stresses in the professional service sector.

### Managerial implications

The findings from Study 1 and Study 2 have pointed to certain behavior control practices capable of improving ethical behaviors in the professional service sector. First, professional organizations need to realize that behavior control practices do make employees behave ethically. Operational managers should encourage employees to exercise caution by adopting an incremental attitude while performing routinized and customized work to avoid risky decision-making. Also, organizations can use formal control procedures. Organizations should design more detailed and specific work procedures to guide professionals, thereby reducing their operational discretion. In addition, they need to consider the importance of information system intensity. The frequency and richness of information system monitoring, evaluating, and dealing with problems in service processes can be used to curb professionals’ risky behaviors, thereby encouraging them to perform ethically. In summary, we suggest that managers can prioritize their investment in behavior practices to improve employee ethical behaviors, reducing the ethical scandals.

Second, professional organizations adopting behavior control practices need to consider the negative impacts of role stress, especially of role ambiguity and role overload. When organizations adopt the practices emphasizing risk control and information system intensity, they need to be clear about the task requirements and expectations of professionals. When service organizations adopt the practices of process control formality, they need to reduce task overloads and pressures on their professionals. In all, in the professional service sector (e.g., banking), it is better to adopt the practices of risk control and information system intensity because it is difficult for such organizations to reduce their employees’ task overload and pressure.

Third, governments or relevant institutions should issue some regulations about operational behavior for these professional companies, for example, the practices about risk control, to help professions to be clear about the task requirements and expectations of professionals. Also, relevant government departments should provide some supports for information system establishment to further avoid the behavior risks of the professionals. When implementing such strict risk management mechanism, governments should appeal more professional companies to reduce the role stress for employees and develop the relax work climate.

### Limitations and future work

This research points to several limitations as possible topics for future research. First, the total reliance cross-sectional data may have influenced the measurement accuracy of the variables such as ethical performance and role stress. Future researchers may use some longitudinal data to test our models more accurately. For instance, they can collect data on behavior control practices in three consecutive years along with data on the variables of ethical behaviors. That should help test the models robustly. Second, we have confined our study to the banking industry. Future studies can enrich the sample types, for example, by including other types of professional services such as health care and law offices, to test whether different types of professional services have different effects. Third, our Study 2 only examined the effective practices from our Study 1. Further study could offer more insights about whether other practices (e.g., employee education and training) have limited effectiveness by using filed experiments. Finally, the research setting of our work was China. Although control practices in China are fairly similar to those in other countries, one can expect ethical problems to be influenced by culture and institutions. In future studies, it is worthwhile to consider some contextual factors relevant to ethical behaviors.

## Data availability statement

The raw data supporting the conclusions of this article will be made available by the authors, without undue reservation.

## Ethics statement

Ethical review and approval was not required for the study on human participants in accordance with the local legislation and institutional requirements. Written informed consent for participation was not required for this study in accordance with the national legislation and the institutional requirements.

## Author contributions

YH, YY, and KCL contributed to conception, design of the study, and wrote sections of the manuscript. YY and KCL organized the database. YY performed the statistical analysis. All authors contributed to manuscript revision, read, and approved the submitted version.
